# Long-term Outcomes of Transoral Outlet Reduction (TORe) for Dumping Syndrome and Weight Regain After Roux-en-Y Gastric Bypass

**DOI:** 10.1007/s11695-023-06466-w

**Published:** 2023-01-27

**Authors:** Valerio Pontecorvi, Maria Valeria Matteo, Vincenzo Bove, Martina De Siena, Giulia Giannetti, Giorgio Carlino, Giulia Polidori, Laila Vinti, Giulia Angelini, Amerigo Iaconelli, Pietro Familiari, Marco Raffaelli, Guido Costamagna, Ivo Boškoski

**Affiliations:** 1 Digestive Endoscopy Unit, Fondazione Policlinico Universitario Agostino Gemelli IRCCS, Largo A. Gemelli, 8 00168, Rome, Italy; 2 Centre for Endoscopic Research Therapeutics and Training (CERTT), Università Cattolica del Sacro Cuore, 00168, Rome, Italy; 3 Università Cattolica del Sacro Cuore, 00168, Rome, Italy; 4 Bariatric Medicine Unit, Fondazione Policlinico Universitario Agostino Gemelli IRCCS, 00168, Rome, Italy; 5 Endocrine and Metabolic Surgery Unit, Fondazione Policlinico Universitario Agostino Gemelli IRCCS, 00168, Rome, Italy

**Keywords:** Obesity, Dumping syndrome, Weight regain, Gastric bypass, Bariatric endoscopy, Transoral outlet reduction

## Abstract

**Background:**

Both weight regain and dumping syndrome (DS) after Roux-en-Y gastric bypass (RYGB) have been related to the dilation of gastro-jejunal anastomosis. The aim of this study is to assess the safety and long-term efficacy of endoscopic transoral outlet reduction (TORe) for DS and/or weight regain after RYBG.

**Materials and Methods:**

A retrospective analysis was performed on a prospective database. Sigstad’s score, early and late Arts Dumping Score (ADS) questionnaires, absolute weight loss (AWL), percentage of total body weight loss (%TBWL), and percentage of excess weight loss (%EWL) were assessed at baseline and at 6, 12, and 24 months after TORe.

**Results:**

Eighty-seven patients (median age 46 years, 79% female) underwent TORe. The median baseline BMI was 36.2 kg/m^2^. Out of 87 patients, 58 were classified as “dumpers” due to Sigstad’s score ≥ 7. The resolution rate of DS (Sigstad’s score < 7) was 68.9%, 66.7%, and 57.2% at 6, 12, and 24 months after TORe, respectively. A significant decrease in Sigstad’s score as well as in early and late ADS questionnaires was observed (*p* < 0.001). The median Sigstad’s score dropped from 15 (11–8.5) pre-operatively to 2 (0–12) at 24 months. The %TBWL was 10.5%, 9.9%, and 8.1% at 6, 12, and 24 months, respectively. Further, “dumpers” with resolution of DS showed better weight loss results compared with those with persistent DS (*p* < 0.001). The only adverse event observed was a perigastric fluid collection successfully managed conservatively.

**Conclusion:**

TORe is a minimally invasive treatment for DS and/or weight regain after RYGB, with evidence of long-term efficacy.

**Graphical Abstract:**

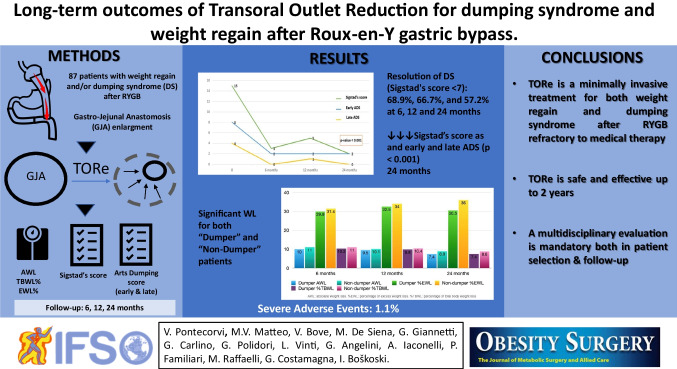

**Supplementary Information:**

The online version contains supplementary material available at 10.1007/s11695-023-06466-w.

## Introduction

Roux-en-Y gastric bypass (RYGB) is one of the most frequently performed bariatric surgeries and has proved excellent long-term outcomes in terms of weight loss and comorbidity improvement [1–3]. However, about one-third of patients could experience weight regain over time and the onset of long-term adverse events, including dumping syndrome (50–70%) [4–9].

Dumping syndrome (DS) consists of a cluster of symptoms induced by the rapid transit of undigested food into the small bowel. The dilation of the gastro-jejunal anastomosis has been related to DS, along with weight regain after RYGB [10, 11]. According to the onset of clinical manifestations, DS can be classified in early and late. The first therapeutic step should be non-invasive with patient education about dietary adjustments and oral therapy [6, 12, 13]. The traditional approaches to patients with intractable DS are surgical revision or continuous enteral nutrition, which have limited efficacy and non-negligible risks [6, 14–16]. Several endoscopic techniques for the revision of the dilated gastro-jejunal anastomosis have been proposed as a minimally invasive treatment for weight regain and, more recently, for DS after RYBG with promising results [10, 17, 18].

The aim of this study is to assess the safety and long-term efficacy of endoscopic transoral outlet reduction (TORe) for DS and weight regain after RYBG in a single tertiary center.

## Methods

### Study Design, Ethics, and Participants

A retrospective analysis was performed on a prospective database collecting data on patients who underwent TORe between January 2015 and June 2021 at the Digestive Endoscopy Unit of Fondazione Policlinico Universitario A. Gemelli IRCCS in Rome. The institutional ethical committee approved this clinical investigation (register no. 19201/18, ID 2082). Informed consent was obtained from all individual participants included in the study. The study was performed in accordance with the ethical standards as laid down in the 1964 Declaration of Helsinki and its later amendments or comparable ethical standards.

The inclusion criteria were:

- Weight regain ≥ 50% of the weight loss after RYGB
- DS refractory to medical therapy
- Endoscopic evidence of gastro-jejunal anastomosis
- Pre-operative assessment by local bariatric multidisciplinary team with indication to endoscopic revision.

All patients who did not fulfil inclusion criteria and those with other types of bypass (i.e., one anastomosis gastric bypass, functional laparoscopic RYGB with fundectomy and gastric remnant exploration) were excluded from the analysis.

Dumping syndrome’s presence and severity were assessed by Sigstad’s score (Table 1) [19]. The score assigns points to symptoms of dumping elicited by a carbohydrate-rich meal. Patients were defined as “dumper” in case of Sigstad’s score ≥ 7. To further evaluate the severity of both early and late DS, we used the Arts Dumping Score (ADS) questionnaire (Table 2) [20]. The questionnaires were obtained at baseline and 6, 12, and 24 months after TORe. Data about weight loss were also collected at baseline and during follow-up. Absolute weight loss (AWL), expressed in kilograms (kg), was calculated as weight before the procedure − weight after the procedure. Percentage of total body weight loss (%TWL) was expressed as follows: ([baseline weight − post-procedure weight]/[baseline weight]) × 100. Percentage of excess body weight loss (%EWL) was expressed as follows [(baseline weight − post-procedure weight)/(baseline weight − ideal weight)] × 100. Ideal body weight was estimated according to BMI 25 kg/m^2^.

**Table 1:** Sigstad’s scoring system

Symptoms	Score
Shock	+ 5
Fainting (syncope), unconsciousness	+ 4
Desire to lie or sit down	+ 4
Breathlessness (dyspnea)	+ 3
Weakness, exhaustion	+ 3
Sleepiness, drowsiness, apathy, falling asleep	+ 3
Palpitation	+ 3
Restlessness	+ 2
Dizziness	+ 2
Headaches	+ 1
Feeling of warmth, sweating, pallor, clammy skin	+ 1
Nausea	+ 1
Abdominal fullness, meteorism	+ 1
Borborygmus	+ 1
Eructation	− 1
Vomiting	− 4

A score ≥ 7 is highly suggestive of dumping syndrome

A score < 4 suggests considering other diagnoses

**Table 2:** Arts Dumping Score questionnaire

Early dumping syndrome symptoms	Late dumping syndrome symptoms
Sweating	Sweating
Flushing	Palpitations
Dizziness	Hunger
Palpitations	Drowsiness and/or unconsciousness
Abdominal pain	Tremor
Diarrhea	Irritability
Bloating	
Nausea	

For each symptom: 0 = absent, 1 = mild, 2 = relevant, and 3 = severe

### Endoscopic Procedure

**Fig. 1:**
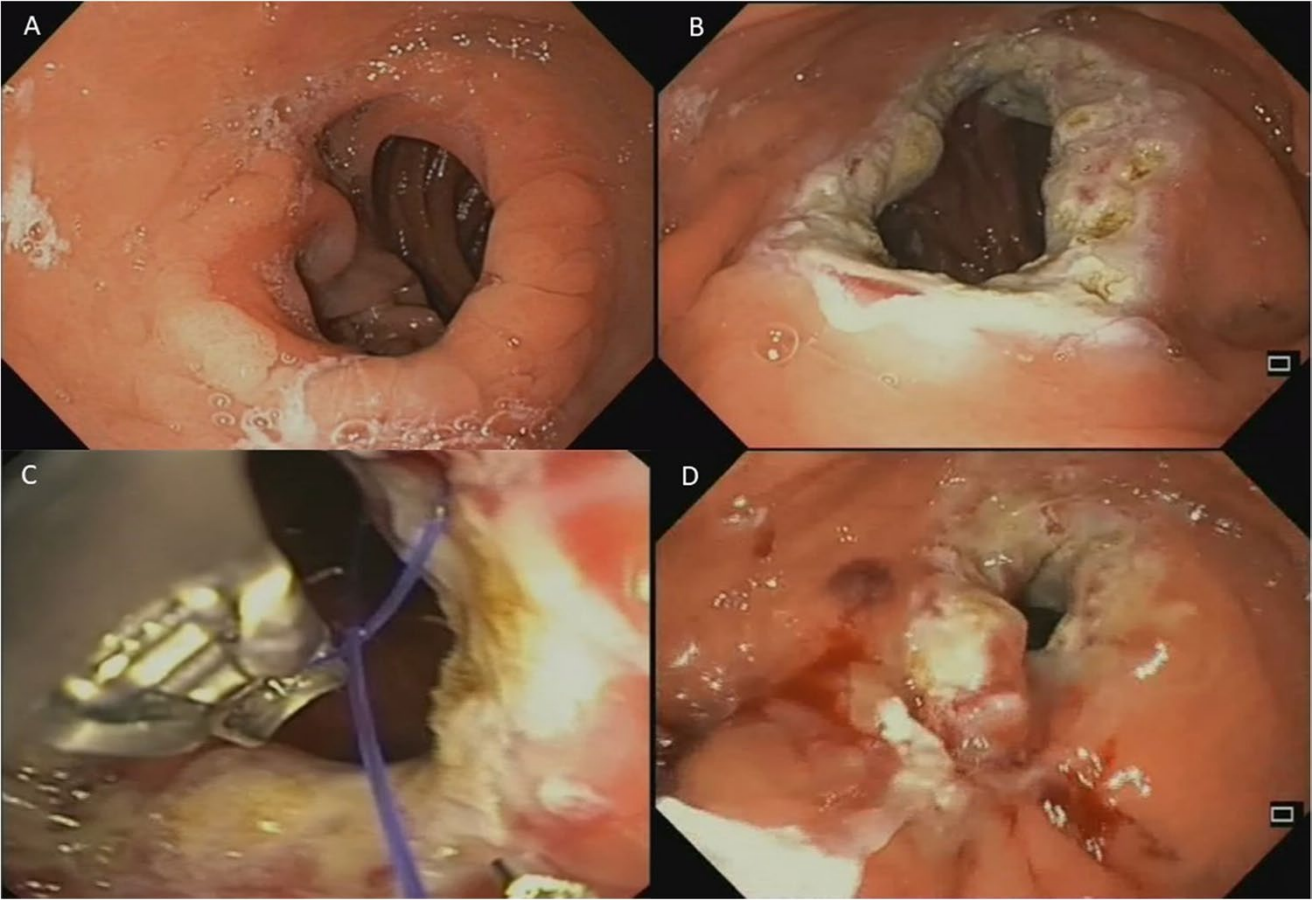
Transoral outlet reduction of the gastro-jejunal anastomosis in RYGB. **A** Endoscopic appearance of the enlarged gastro-jejunal anastomosis. **B** Gastro-jejunal anastomosis after APC. **C** Suturing of the GJA. **D** Final appearance of the endoscopic reduction

All TORe procedures were performed under general anesthesia using the full-thickness suturing system Apollo OverStitch (Apollo Endosurgery, Austin, TX, USA), and a double-channel therapeutic endoscope (GIF 2HT180 or GIF 2HT160; Olympus, Spring Valley, PA, USA). Before suturing, the rim of the anastomosis was cauterized by argon plasma coagulation (APC) at a flow of 1 L/min and 40 W to mark the margins of the anastomosis and to enhance the suture tightening during the scarring process (Fig. 1A–B, Video 1). An interrupted suturing technique with two bites for each suture was used. A total of 2–3 sutures were placed for each patient, obtaining a reduction of the anastomosis’ diameter of about 80% (Fig. 1C–D, Video 1). At the end of the procedure, the absence of complications was checked with a standard gastroscope. After TORe, patients followed a liquid diet on the first postoperative day and were discharged after 24 h. Each patient was provided with a personalized 6-week dietary plan, including a gradual shift from a liquid to a solid diet. After the procedure, follow-up visits were scheduled at 1, 6, 12, and 24 months, as per routine clinical practice. In case of impossibility of attending the visit in-person, phone calls were made.

### Statistical Analysis

Statistical analyses were conducted using the STATA version 16 software (STATA Corp). All variables included in the study were summarized using descriptive statistical techniques. Qualitative data were reported as absolute and percentage frequencies. As regards the quantitative variables, the distribution of the data was first verified by the Shapiro-Wilk normality test. Continuous variables were reported as means and standard deviations (SD) or as medians and interquartile ranges (IQR) in case of deviations from normality. The differences between baseline (TORe) and 6, 12, and 24 months were evaluated, as regards the quantitative variables, both overall, through the non-parametric Friedman test, and two by two (0–6 months, 6–12 months, and 12–24 months) through the non-parametric Wilcoxon test for paired samples. Comparisons between qualitative variables, pre-post according to the above scheme, were performed by McNemar’s test. Comparison in weight loss parameters in patients with resolution of DS and patients with refractory DS after treatment was performed by the non-parametric Wilcoxon test. A *p*-value < 0.05 was considered statistically significant.

## Results

Eighty-seven patients underwent TORe between January 2015 and June 2021 at our center. Baseline characteristics of the patients are summarized in Table 3.

**Table 3:** Baseline characteristics of patients (*n* = 87)

Age (years)	46 (39–52)
M/F, *n (%)*	18 (20.7%)/69 (79.3%)
Before RYGB	
Weight (kg)	133 (120–145)
Height (cm)	164 (160–170)
BMI (kg/m^2^)	48.4 (44.5–51.9)
Time between RYGB-TORe (months)	83 (51–108)
Before TORe	
Weight (kg)	97 (87–112)
BMI (kg/m^2^)	36.2 (31.9–39.8)
EW (kg)	29.3 (19.4–39.8)
“Dumpers” patients, *n* (%)	58 (66.7)
Sigstad’s score	15 (11–18.5)
EADS	8 (5.5–11)
LADS	4 (2–7)

Values are median (IQR) unless otherwise defined

*TORe*, transoral outlet reduction; *M,* male; *F*, female; *IQR*, interquartile range; *RYGB*, Roux-en-Y gastric bypass; *BMI*, body mass index; *EADS*, early Arts Dumping Score questionnaire; *LADS*, late Arts Dumping Score questionnaire

All 87 patients (100%) completed the 6-month follow-up. Seventy-six patients (87.4%) completed the 12-month follow-up, 3 (3.4%) were lost to follow-up, and 7 (8%) were still waiting to complete the 12-month follow-up. Fifty-six patients (64.4%) completed the follow-up at 24 months, 7 (8%) were lost to follow-up, and 22 (25.3%) had yet to reach the 24-month follow-up. After a multidisciplinary assessment, TORe was repeated in one previously “non-dumper” patient due to the new onset of DS within 12 months and in two other “non-dumper” patients (2.3%) because of weight regain within 2 years. In total, 3 patients required a re-TORe and were excluded from the statistical analysis. To provide a scientifically robust evaluation of the variation over time of dumping syndrome’s parameters and weight loss outcomes after TORe, we focused statistical analyses on those patients who completed the follow-up at 24 months.

## Safety

One serious adverse event was observed: a perigastric collection, in a patient with a clinical history of post-traumatic splenectomy, occurred in the first 48 h. The patient complained persistent post-procedural abdominal pain and fever and was successfully treated with piperacillin-tazobactam (grade II sec. Clavien-Dindo classification). No procedure-related deaths and no other adverse events were observed.

## Dumping Syndrome Outcomes

**Fig. 2:**
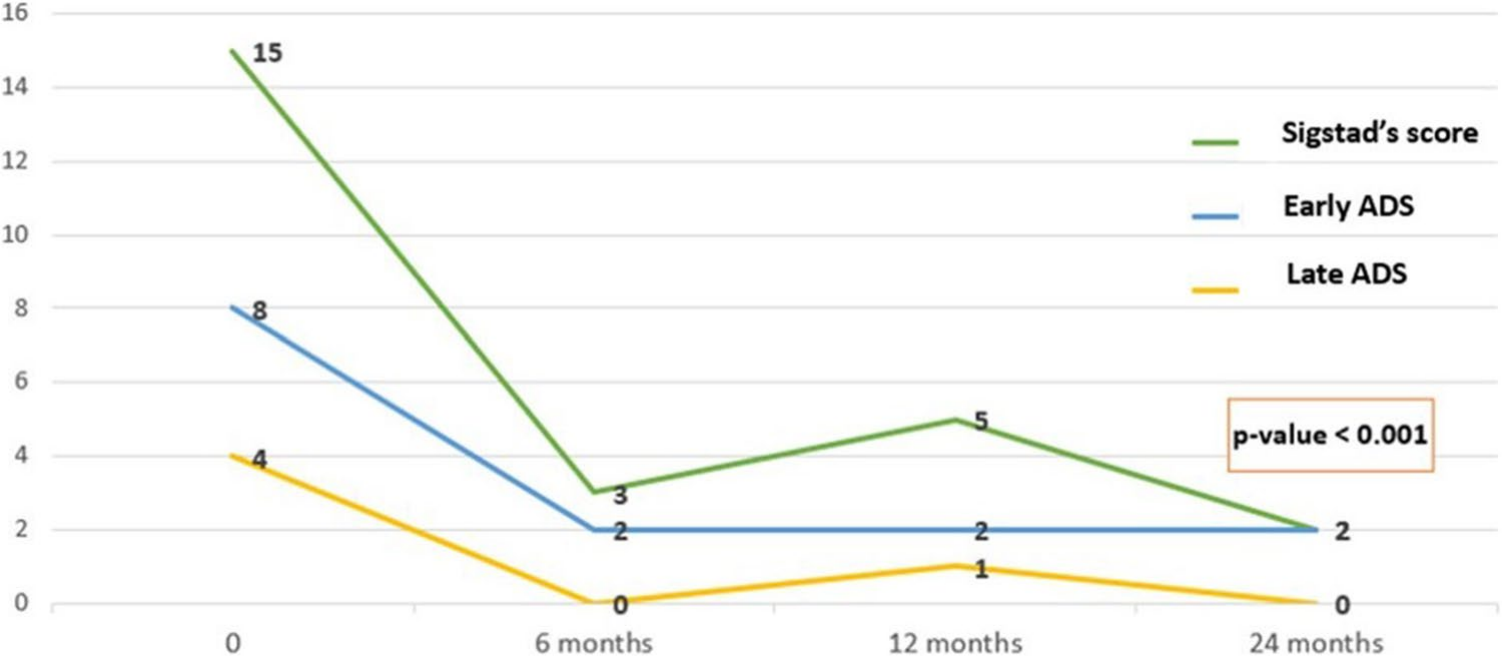
Trend of symptom-based scores for dumping syndrome after TORe

Fifty-eight out of 87 patients (66.7%) were classified as “dumpers” at baseline according to Sigstad’s score ≥ 7. Of these, 51 reached follow-up at 12 months and 35 reached follow-up at 24 months. Forty of 58 patients (68.9%), 34 of 51 patients (66.7%), and 20 of 35 patients (57.2%) showed resolution of symptoms (Sigstad’s score) < 7 at 6, 12, and 24 months, respectively. We observed a statistically significant decrease in all symptom-based scores for DS (Table 4, Fig. 2). The post hoc analysis showed that the difference is statistically significant between baseline compared with each follow-up (6–12–24 months), while there is no significant difference in multiple comparisons between 6–12 and 12–24 months, which are therefore superimposable.

**Table 4:** Dumping syndrome outcomes (*n* = 35*)

	Baseline	6 months	12 months	24 months	*p*^*#*^
Sigstad’s score	15 (11–18.5)	3 (1–9.5)	5 (1–12)	2 (0–12)	< 0.001
EADS	8 (5.5–11)	2 (0–5)	3 (1–6.5)	2 (0–4.5)	< 0.001
LADS	4 (2–7)	0 (0–2)	1 (0–3)	0 (0–2)	< 0.001

*Dumper patients who reached the 24-month follow-up

Values are median (IQR) unless otherwise defined

*EADS*, early Arts Dumping Score questionnaire; *LADQ*, late Arts Dumping Score questionnaire

^#^Friedman’s test

## Weight Loss Outcomes

**Fig. 3:**
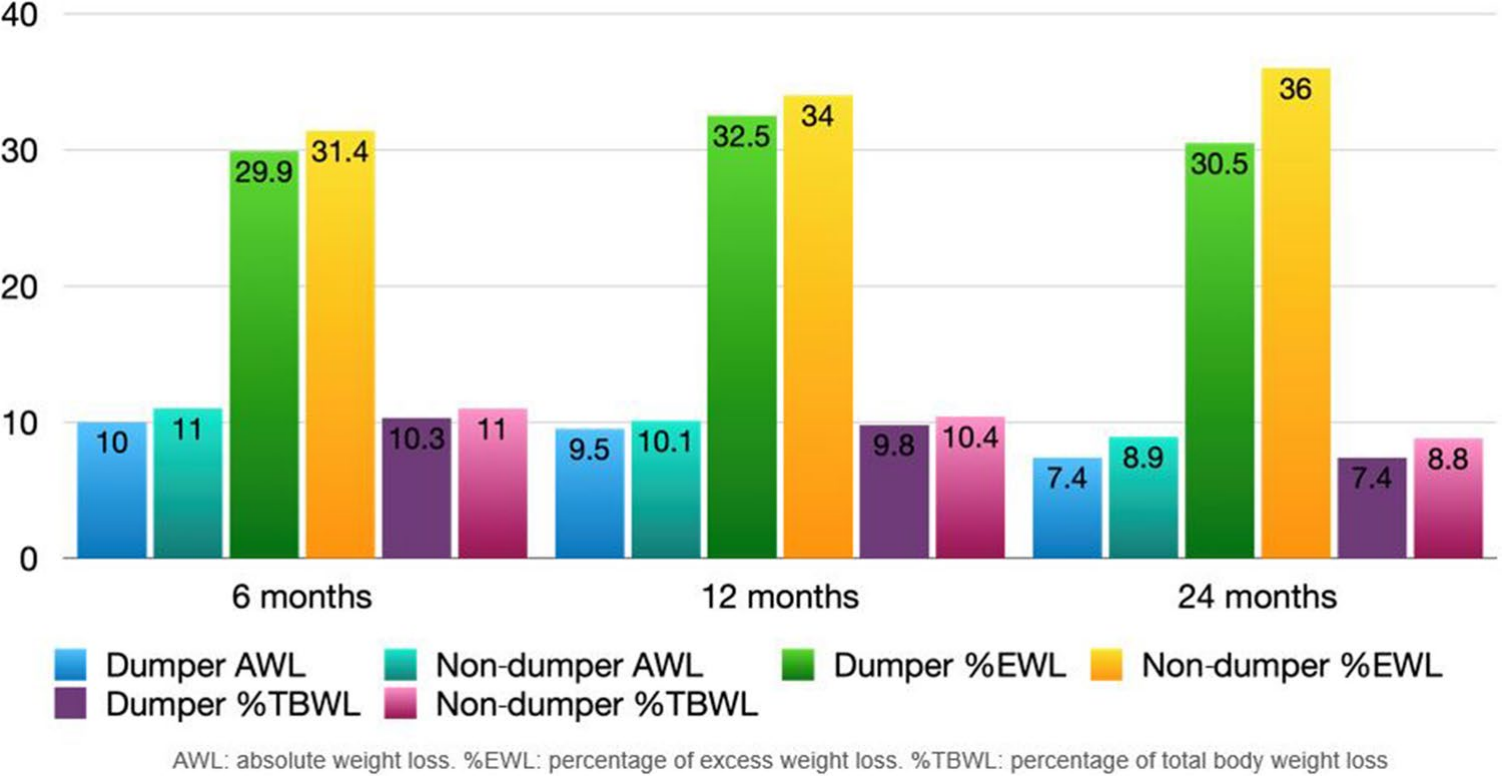
Weight loss outcomes in “dumper” patients and “non-dumper” patients according to indication to TORe at baseline

**Fig. 4:**
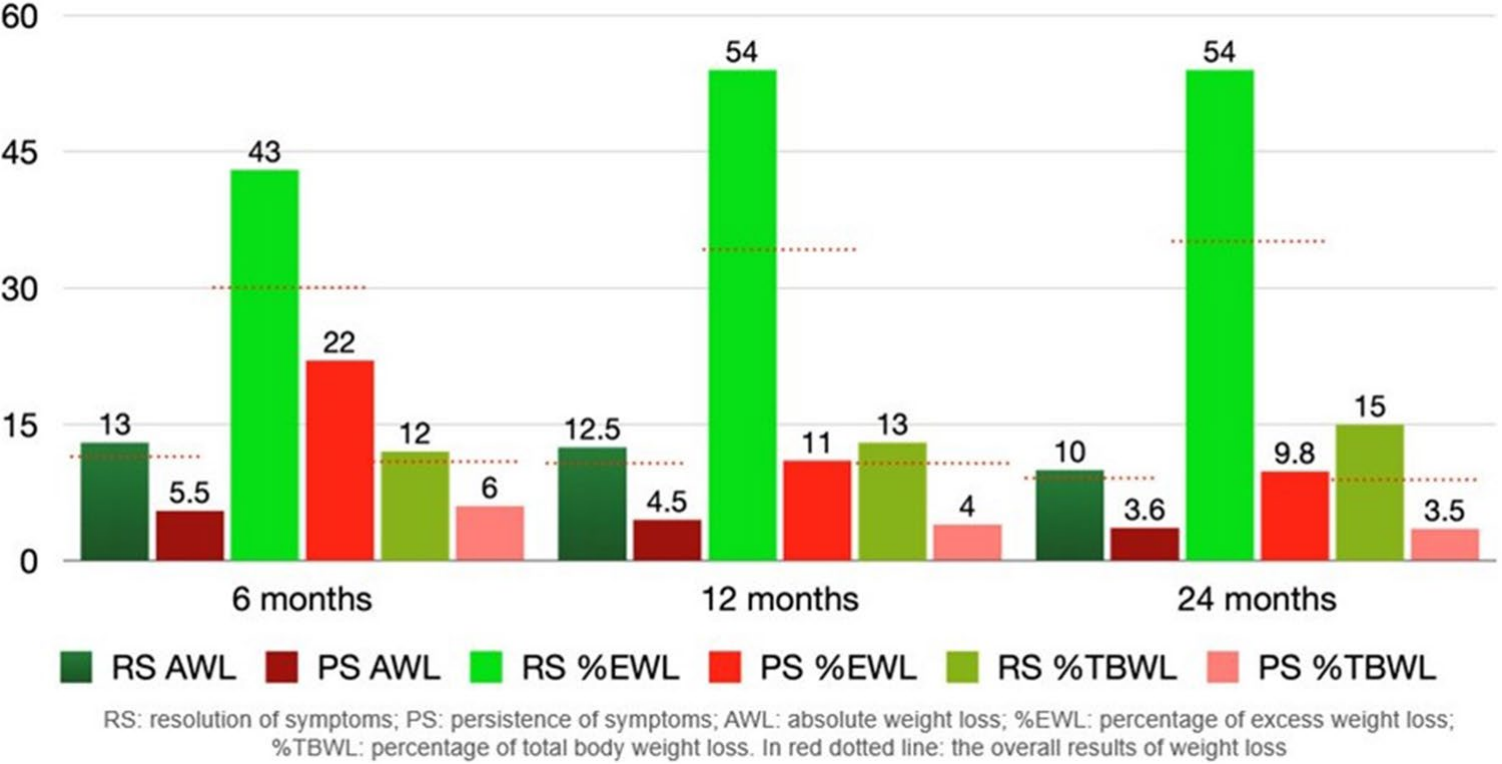
Weight loss outcomes in “dumper” patients at baseline according to resolution or persistence of dumping syndrome

Table 5 has summarized weight loss outcomes after TORe. The post hoc analysis showed a statistically significant difference between baseline compared with each follow-up (6–12–24 months), while no significant difference in multiple comparisons between 6–12 and 12–24 months was detected. Notably, about 61% of patients showed %TBWL > 5% at 24 months. Comparing the two cohorts, “dumper” and “non-dumper” patients, there was no significant difference in terms of AWL, EWL, and TBWL over time (Fig. 3). However, comparing weight loss results between “dumper” patients with resolution of symptoms and “dumper” patients with refractory symptoms after TORe, there were statistically significant differences between the two groups. Patients with resolution of DS had better results on weight loss compared with patients with persistent DS (Fig. 4).

**Table 5:** Overall weight loss outcomes (*n* = 56)

	6 months	12 months	24 months	*p*^*#*^
AWL (kg)	11 (4–14)	10 (1–15)	8 (3–14)	< 0.001
EWL (%)	30.2 (14–44.8)	33.7 (3.8–48.7)	34.2 (9.9–57.8)	< 0.001
TBWL (%)	10.5 (4.1–13.7)	9.9 (1.1–14.3)	8.1 (3.1–13.3)	< 0.001

*AWL*, absolute weight loss; *EWL*, excess weight loss; *TBWL*, total body weight loss

^#^Friedman’s test

## Discussion

Despite the rapid and substantial weight loss observed in the short and medium term after RYBG, a significant subset of patients (one-third) may experience a progressive weight regain over the long term [5, 21, 22]. Failure of sustained weight loss after bariatric surgery as well as the onset of DS has been associated with the dilation of the gastro-jejunal anastomosis [22–25]. Dumping syndrome consists of multiple clinical manifestations elicited by the rapid movement of ingested food from the stomach into the small bowel [6]. Early DS, characterized by gastrointestinal and vasomotor symptoms, arises within 60 min after a meal, while late DS is characterized by hypoglycemic manifestations, arising 1–3 h after a meal [6, 26].

The first step in the management of DS is dietary adjustments, with a reduced quantity of food at each meal and avoidance of simple sugars [6]. As next step, oral therapy with supplements that increase the viscosity of food and medications, such as acarbose and somatostatin analogues, can be considered [6]. Surgical interventions (i.e., stomal revision, Billroth II to Billroth I anastomoses, Roux-en-Y conversion) are the traditional choices for patients with refractory DS [26]. Revisional surgery for DS as well as for weight regain can be technically challenging in the presence of altered anatomy and adhesions and is associated with an increased risk of adverse events, morbidity, and mortality [6, 18, 26].

The endoscopic transoral outlet reduction (TORe) is proposed as a minimally invasive treatment for patients with DS refractory to medical therapy and/or weight regain after RYGB [17, 18]. The rationale for this procedure is to reduce the diameter of the anastomosis, thus delaying the transit of ingested food and prolonging satiety [17, 18]. A multicenter study including 115 patients showed a statistically significant reduction of Sigstad’s score at 3 months after TORe (2.55 ± 1.87 vs 17.23 ± 5.9, *p* = 0.001) [10]. Similarly, Tsai et al. found a short-term improvement in Sigstad’s score (8.6 at 3 months vs 13.9 at baseline) in 90% of the patients (33/37) [25]. Our study showed a good resolution rate of DS after TORe in the medium and long term. The resolution of DS (Sigstad’s score < 7) occurred in 68.9% (40/58), 66.7% (34/51), and 57.2% (20/35) of the “dumper” patients at 6, 12, and 24 months, respectively. We observed a significant decrease in Sigstad’s score (< 7, *p*-value < 0.001) as well as in both early and late ADS questionnaires (*p*-value < 0.001). Eventually, we had no case of recurrence of DS in 2 years.

Regarding weight loss outcomes after endoscopic revision of the anastomosis, a recent systematic review reported a mean weight loss of 6.27 kg, sustained up to 24 months, but the %EWL was 19.3% at 6 months and 10.3% at 24 months [27]. Our data showed superior weight loss with %EWL of 30.2% (14–44.8), 33.7% (3.8–48.7), and 34.2% (9.9–5.8) at 6, 12, and 24 months. A reason could be that in this systematic review, several endoluminal devices and techniques were employed, so the weight loss could be altered by the different efficacies of each technique. Our analysis showed %TBWL of 10.5%, 9.9%, and 8.1% at 6, 12, and 24 months, respectively. These results are comparable with the previous study by Kumar et al. showing a %TBWL of 9.6%, 9.5%, and 8.1% at 6, 12, and 24 months, respectively [18]. Further, about 61% of patients who reached 2 years follow-up showed a %TBWL > 5%, consistent with a percentage of 67% at 1 year reported by Vargas et al. [28]. This threshold is especially relevant since it is associated with a significant obesity-related comorbidity improvement [28, 29].

Further, in our analysis, the two-by-two comparisons (0–6 months, 6–12 months, and 12–24 months) of both symptom-based scores for DS and weight loss outcomes showed no statistical difference between 6–12 and 12–24 months; this evidence suggests that the results obtained in the first months are maintained over 2 years. We did not observe any statistically significant difference in terms of weight loss comparing “dumper” and “non-dumper” patients. Interestingly, we found a statistically significant difference (*p*-value < 0.001) analyzing weight loss outcomes in “dumper” patients with resolution of DS compared to those with persistent symptoms after TORe. In more details, “dumper” patients that resolved DS showed overall better weight loss results compared to those with persistent DS. Probably, the resulting delayed emptying and the smaller amount of food to the small bowel also caused a change in their eating habits. We observed that patients with persistent DS, contrary to what was expected, often tend to eat sugars to mitigate the hypoglycemic symptoms.

One serious adverse event (1.1%) was observed, a perigastric collection that was effectively treated with antibiotic therapy. No procedure-related deaths occurred. TORe is a low-risk procedure, as highlighted in previous studies, where serious adverse events reported are < 1% [10, 17, 18, 26, 27]. Indeed, our percentage is slightly higher, affected by the sample size. To note, this event occurred within the first 5 procedures performed, so the growing technical expertise probably improved the safety of the procedure.

This study has some limitations that should be pointed out, including the retrospective design, the absence of a control group, and a possible selection bias resulting from the single-center counselling. Eventually, data at 2 years were available for only 64.4% of the treated patients. Nevertheless, as strengths, this is the first study evaluating not only the Sigstad’s score but also the Arts Dumping Score as tools to monitor the efficacy of TORe for DS. In more details, the impact of the procedure was analyzed by differentiating between early and late symptoms. Furthermore, this study has a longer follow-up compared with other studies in the literature.

## Conclusion

**Fig. 5:**
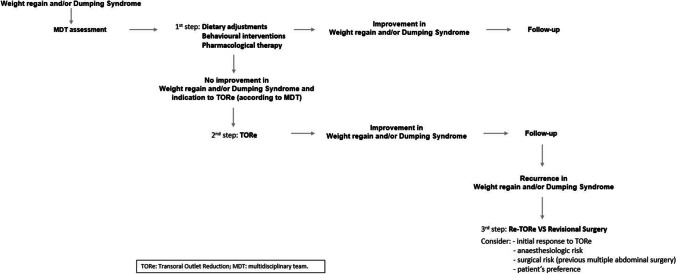
Flowchart: steps in the treatment of weight regain and dumping syndrome after Roux-en-Y gastric bypass

According to our experience, TORe is an effective minimally invasive treatment for both early and late DS along with weight regain after RYGB, with evidence of long-term maintenance of the results achieved in the first months. Given the good outcomes and the excellent safety profile, TORe may play a key role in the multidisciplinary approach to weight regain and/or DS after RYGB when conservative therapy fails and before surgical revision (Fig. 5).

## Supplementary Information

ESM 1: (MP4 108300 kb)
